# Editorial: Dyslipidemia, obesity and coronavirus disease 2019 (COVID-19)

**DOI:** 10.3389/fnut.2022.1019970

**Published:** 2022-11-23

**Authors:** Timotius Ivan Hariyanto, Andree Kurniawan, Dicky Levenus Tahapary

**Affiliations:** ^1^Faculty of Medicine, Pelita Harapan University, Tangerang, Indonesia; ^2^Department of Internal Medicine, Faculty of Medicine, Pelita Harapan University, Tangerang, Indonesia; ^3^Division of Endocrinology and Metabolic Disorders, Department of Internal Medicine, Faculty of Medicine, University of Indonesia, Jakarta, Indonesia

**Keywords:** obesity, dyslipidemia, COVID-19, metabolic disease, nutrition-clinical, endocrinology, SARS-CoV-2

Almost 3 years after its emergence in December 2019, coronavirus disease 2019 (COVID-19) still causes a significant burden on health, economic, and social aspects of life (1). While some SARS-CoV-2 infections appear as mild upper respiratory symptoms and may be self-limiting, there is still a notable number of patients who require hospitalizations and intensive treatment following progression into more severe cases, varying from simple lower respiratory tract infections to acute respiratory distress syndrome (ARDS) or multi-organ failure (MOF). Identification of risk factors for severe disease and special populations at risk are important to help mitigate the pandemic, reducing the morbidity and mortality from COVID-19 (2). Among the comorbidities which are associated with severe COVID-19, dyslipidemia and obesity may become important as these two have often been under-looked. Moreover, the role of drugs commonly used in patients with dyslipidemia and obesity has gathered a lot of attention because they may potentially alter the course of COVID-19. This Research Topic, focused on the role of dyslipidemia, obesity, and their related disorders in the course of COVID-19, consists of a set of eight papers—three original research articles, two brief research reports, two mini-reviews, and one review article.

Countries around the world have implemented strategies to control the COVID-19 pandemic, such as social distancing, lockdown, isolation, quarantine, and so on. However, these measures are increasingly impacting all classes of people with sheer frustration, especially university students, with far-reaching effects on their mental, physical, and social lives. A cross-sectional study conducted in Bangladesh has revealed that during the COVID-19 lockdown, there is an increased prevalence of overweight/obesity among university students due to changes in eating behaviors and reduction in physical activities (Hossain et al.). This is concerning because studies have shown that obesity may increase the risk of mortality and severe COVID-19 complications, including ICU admission and intubation. People with higher body mass index (BMI) also tend to be more vulnerable to SARS-CoV-2 infections (Vassilopoulou et al.). Not only BMI, but also the percentage of body fat (%BF) and visceral fat (%VF) were significantly associated with poor outcomes from COVID-19 (Stevanovic et al.). Therefore, measurement of BMI, %BF, and %VF through bioelectrical impedance analysis (BIA) may serve as a potential tool to predict which patients are at high risk of developing poor COVID-19 outcomes.

People with obesity are suggested to focus on lowering their body weight to achieve a normal BMI target. One of the non-pharmacological treatments which can be offered is dietary changes. Among several available dietary patterns, the ketogenic diet may give benefit not only for healthy subjects but also patients with respiratory diseases, including SARS-CoV-2 infection (Gangitano et al.). This beneficial effect has been attributed to the anti-inflammatory, anti-viral, and weight-reducing effects of a ketogenic diet, which therefore may be considered in obese patients as a preventive measure for COVID-19.

The management of obesity should not only focus on BMI. Several vitamin and mineral concentrations may be altered in people with obesity. The presence of obesity and higher visceral fat content is related to a higher incidence of vitamin D deficiency, probably because of vitamin D sequestration into adipose tissue. A higher proportion of patients with obesity have low vitamin D levels (<20 ng/ml or <50 nmol/L). Both low vitamin D levels and vitamin D deficiency (≤12 ng/ml or <30 nmol/L) are associated with higher mortality from COVID-19 independent of BMI, partly mediated by the effect of vitamin D on markers of disease severity, such as D-dimer and ultrasensitive cardiac troponins (Vanegas-Cedillo et al.). Besides vitamin D, magnesium may also be associated with obesity. Magnesium is known to have roles in glucose, insulin, and energy homeostasis through its activity on pancreatic beta-cells, increasing glucose uptake from peripheral tissue, and modulation of the inflammatory process. Magnesium deficiency is frequently observed in obese people. A cross-sectional study in Iran has shown that high magnesium intake in COVID-19 patients was associated with lower levels of several inflammatory biomarkers, such as C-reactive proteins (CRP) and erythrocyte sedimentation rate (ESR; Nouri-Majd et al.). Patients with higher dietary magnesium intake may also have lower odds of developing severe COVID-19 conditions compared to those with low magnesium intake. We can conclude from these studies that vitamin D and magnesium supplementation should be considered to reduce the burden of COVID-19 in those who are deficient, especially people with obesity.

When treating people with obesity, we must also consider its related disorders such as dyslipidemia. Dyslipidemia has been repeatedly shown to be associated with the risk and severity of COVID-19, and one of the mainstay therapies in patients with dyslipidemia is a statin. Studies have shown that statins are generally safe for COVID-19 patients and may even offer therapeutical benefits, owing to their antiviral, immunomodulatory, anti-thrombosis, and anti-oxidative effects (Liu et al.). The use of statins should therefore not be discontinued during COVID-19, especially in those who have dyslipidemia where the benefit from statins will be more prominent. Besides statins, studies have shown that L-carnitine has a key role in fatty acid metabolism and could function as an additional agent to improve dyslipidemia. Using the Mendelian randomization method, it has been demonstrated that carnitine might have a protective role on COVID-19, and carnitine might be a therapy that is worth further exploration in clinical trials (Li et al.).

We hope that the studies under our Research Topic collection may give us a better understanding of COVID-19 and its relationship with both obesity and dyslipidemia. Their results enable better risk stratification, earlier detection, and a more holistic approach for these populations. These publications also show how this field is in an emergent phase and how much research is needed in this area of knowledge.

## Author contributions

All authors listed have made a substantial, direct, and intellectual contribution to the work and approved it for publication.

## Conflict of interest

The authors declare that the research was conducted in the absence of any commercial or financial relationships that could be construed as a potential conflict of interest.

## Publisher's note

All claims expressed in this article are solely those of the authors and do not necessarily represent those of their affiliated organizations, or those of the publisher, the editors and the reviewers. Any product that may be evaluated in this article, or claim that may be made by its manufacturer, is not guaranteed or endorsed by the publisher.
